# Effects of the Essential Oil from *Pistacia lentiscus* Var. *chia* on the Lateral Line System and the Gene Expression Profile of Zebrafish (*Danio rerio*)

**DOI:** 10.3390/molecules24213919

**Published:** 2019-10-30

**Authors:** Iliana Serifi, Eleni Tzima, Haido Bardouki, Evangeli Lampri, Thomais Papamarcaki

**Affiliations:** 1 Laboratory of Biological Chemistry, Department of Medicine, School of Health Sciences, University of Ioannina, 451 10 Ioannina, Greece; iliana.serifi@gmail.com (I.S.); elenatzima@gmail.com (E.T.); 2 Division of Biomedical Research, Foundation for Research and Technology-Hellas, Institute of Molecular Biology and Biotechnology, 451 10 Ioannina, Greece; 3 VIORYL S.A., Chemical & Agricultural Industry, Research S.A., 28th km Athens-Lamia, RD, 190 14 Afidnes, Greece; bardouki@vioryl.gr; 4 Department of Pathology, Department of Medicine, School of Health Sciences, University of Ioannina, Ioannina, 451 10 Ioannina, Greece; evangeli.lampri@gmail.com

**Keywords:** mastic essential oil, zebrafish, lateral line system, gene expression, microbiota, natural products

## Abstract

Mastic essential oil exhibits anti-bacterial, anti-inflammatory, and anti-oxidant properties. With the growing interest of the use of mastic oil in the food and pharmaceutical industry, systematic in vivo studies are needed to address controlled usage and safety issues. In the present work we evaluated the safety of mastic oil using as a model the zebrafish lateral line system. In addition, we studied the gene expression profile of zebrafish fed with mastic oil-supplemented diet using microarray analysis. Our results showed that the hair cells of lateral line neuromasts are functional upon exposure of zebrafish larvae up to 20 ppm of mastic essential oil, while treatment with higher concentrations, 100 and 200 ppm, resulted in increased larvae mortality. Dietary supplementation of zebrafish with mastic essential oil led to differential expression of interferon response-related genes as well as the immune responsive gene 1 (*irg1*) that links cellular metabolism with immune defense. Notably, *mucin 5.2*, a constituent of the mucus hydrogel that protects the host against invading pathogens, was up-regulated. Our in vivo work provides information concerning the safety of mastic essential oil use and suggests dietary effects on gene expression related with the physical and immunochemical properties of the gastrointestinal system.

## 1. Introduction

Mastic is a natural resin obtained from the plant *Pistacia lentiscus* variation *chia* which is cultivated in the island of Chios, in the Eastern Aegean Sea. It has been known for centuries that chewing mastic gum assists in mouth disinfection and alleviates oral malodour. Recent research has provided scientific evidence to support this traditional knowledge, highlighting the importance of this natural product for human’s health [1,2,3,4]. Mastic has been used in medicine since antiquity and its antimicrobial and antifungal activities have been intensively exploited by the pharmaceutical/medicinal industry. Mastic fights *Helicobacter pylori* bacteria and cures peptic ulcers even when administered in small doses [5,6,7,8]. Interestingly, previous work has shown that mastic prevents the oxidation of LDL and decreases the risk of atherosclerosis [9,10], while other studies have shown anti-inflammatory and anti-oxidant properties [11,12]. 

The mastic gum essential oil is the product of the resin’s hydrodistillation and *α*-pinene is its major compound [13,14,15]. Several studies have reported on the anti-bacterial properties of the mastic essential oil against distinct bacterial strains, including *E. coli*, *S. aureus,* and *B. subtilis* [13,16]. Mastic essential oil has also anti-tumour growth activities against several cancers such as leukemia, adenocarcinoma, colon, prostate, and lung cancer [17,18,19,20,21,22], but the mechanisms by which it exerts the above activities are still not understood. It has been postulated that exposure to mastic essential oil affects directly or indirectly signaling pathways that modulate gene expression, cell proliferation, and differentiation.

Until now, studies regarding the effects of mastic essential oil on human health are limited, since experimental strategies have been based mostly on cell culture systems. Considering the growing interest in the use of mastic oil in the food, cosmetic, and pharmaceutical industry, organism-level systematic studies are needed to evaluate its potential effects and safety usage. Zebrafish is an excellent model for developmental and toxicological studies that can be directly compared to other vertebrates, including mammals. Currently, zebrafish is a well-established in vivo experimental system for investigating host microbial-chemical interactions and innate immune responses [23]. During early development, the survival of zebrafish larvae is dependent solely on the innate immune responses because the adaptive immune system is functionally mature after 4–6 weeks post fertilization [24]. Recently, the anti-inflammatory effects of essential oils extracted from *Thymus vulgraris* and *Rosmarinus officinalis L*. have been studied in zebrafish larvae [25,26]. Furthermore, zebrafish has been recently used in fish nutrition studies such as dietary supplementation with l-carnitine and microalgae-derived arachidonic acid [27,28].

In our work we investigated the effects of mastic essential oil on (i) the zebrafish lateral line sensory system and (ii) the gene expression profile of zebrafish juveniles in response to mastic oil dietary supplementation. The zebrafish lateral line system is comprised of mechanoreceptive organs, called neuromasts, which are innervated by sensory neurons projecting to the central nervous system. Each neuromast consists of sensory hair cells that are morphologically characterized by the presence of a single microtubule-based cilium (kinocilium) flanked by actin-filled projections (stereocilia) [29]. These structures are highly sensitive to drugs, antibiotics, and environmental compounds [30]. 

Our results indicate that the lateral line system of zebrafish larvae exposed to 10–20 ppm of mastic essential oil, retains its functionality which is suggestive of a natural product with no toxicity at this concentration range. On the contrary, higher concentrations of the essential oil such as 100 and 200 ppm, strongly affected the larval development indicating toxic effects. At equivalent to these high concentrations, its constituent *a*-pinene exhibited no developmental toxicity, but it affected the activity of neuromasts. The diet supplementation of zebrafish juveniles with mastic essential oil, resulted in differential expression of genes related to the immune defense against viruses and bacteria. To our knowledge this is the first study that addresses safety issues and gene expression effects of mastic essential oil within the context of an organism under physiological conditions, emphasizing the potential of the zebrafish model system for future studies.

## 2. Results and Discussion

The resin of Pistacia lentiscus var. chia (mastic gum) has been known and utilised since ancient times in food, flavouring, cosmetics, and medicine, and recently it has been approved as a natural medicine by the European Committee on Herbal Products (HMPC) (July 22, 2015). The mastic essential oil is one of the most popular essential oils and numerous studies have documented a variety of health benefits including anti-bacterial, analgesic, anti-inflammatory, and anti-viral properties [31]. Mastic essential oil was extracted from the resin that was obtained as a trunk exudate from the mastic tree, by direct gum distillation. It is composed of mainly volatile terpenes, with established distinct biological roles. Its unique composition accounts for the multiple uses of mastic oil and its constituents in food industry and healthcare [13,14]. Analysis of mastic oil preparation by GC/MS (Figure S1) identified several components with the most abundant ones being α-pinene (67.7%), myrcene (18.8%), and β-pinene (3.0%) (Table 1).

Essential oils are commonly recognized as safe products with beneficial effects for animals and consumers, although it is well documented that several phytochemicals can potentially interfere with cell pathways and in particular of those in neuronal cells [32]. Concerning mastic essential oil, a small number of in vivo studies exists to date with significant differences in the protocols and dosages used, as well as in the ways of administration [33,34]. Therefore, additional in vivo studies are necessary to evaluate safety issues of mastic essential oil.

**Figure 1:**
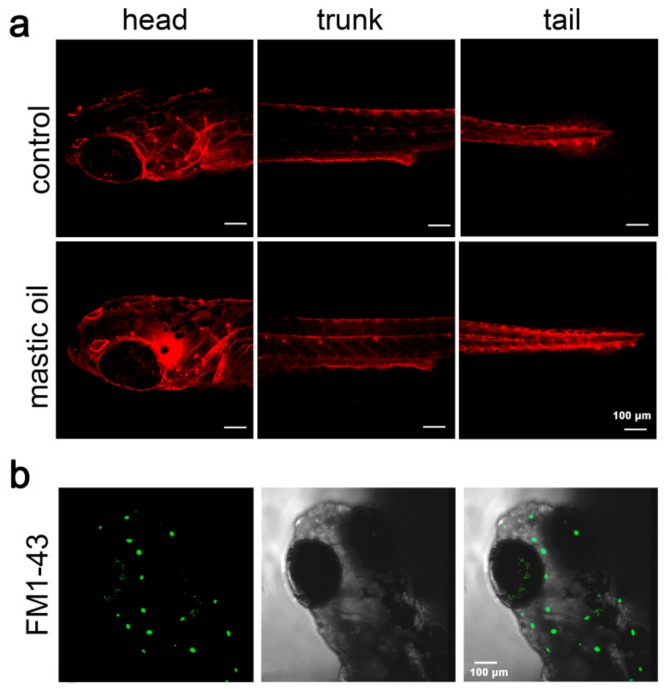
Architecture of the lateral line system in mastic essential oil-treated zebrafish. (**a**) Acetylated tubulin staining of control and mastic oil-treated zebrafish larvae at 7 dpf. (**b**) A representative image showing FM1-43 staining of 7 dpf zebrafish larvae.

Several lines of evidence highlight the zebrafish lateral line as a suitable model to screen for drug and chemical compound safety, since morphological alterations such as malformations or reduction of body length, frequently used as toxicity markers, are observed as late or secondary effects. The lateral line system consists of neuromasts which are superficial sensory organs composed of clusters of hair cells, supporting cells, and integrated neurons [29]. Transcriptome analysis after hair-cell ablation has uncovered genes that are differentially regulated early in regeneration, providing valuable information for hair-cell regeneration studies [35]. This analysis can be combined with a repertoire of larvae behavioral assays to access the integrity and/or functionality of their sensory organs [36]. The neuromast hair cells selectively take up fluorescent vital dyes, facilitating the in vivo assessment of the sensory organs formation and/or activity. Using this in vivo approach, the effects of various natural and synthetic compounds on neuromasts, can be monitored by their addition to the surrounding water at different concentrations. To evaluate potential toxic effects of mastic essential oil, 3 days post fertilization (dpf) zebrafish larvae were treated for four days with 10, 20, 100, and 200 ppm of the mastic oil and larval mortality was first assessed. Our results showed that mastic essential oil at the range of 10–20 ppm did not affect the morphology or the swimming behavior of zebrafish larvae (not shown). Furthermore, the architecture of the lateral line network was normal upon exposure to 20 ppm of mastic oil, as assessed by acetylated tubulin staining (Figure 1a). Higher concentrations of 100 and 200 ppm of mastic essential oil, led to high mortality rates, affecting also the swimming behavior of the larvae which was marginal. 

To examine whether mastic essential oil affects the sensory activity of the lateral line, we assessed the functionality of neuromasts upon exposure of 3 dpf larvae to 20 ppm of mastic oil for four days and subsequent labeling with FM1-43 (Figure 1b). The rapid uptake of this vital dye via the mechano-transduction channels of hair cells indicated that under these conditions the neuromasts remained functional. Labeled neuromasts were counted bilaterally in 25 larvae and the average number was determined to be 51.64 ± 4.4 (S.D) in the mastic oil-treated and 53.36 ± 3.7 (S.D) in the control group. These results suggest that mastic oil does not interfere with the function of zebrafish neuromasts and could be considered as a safe product at this concentration range.

It is generally thought that the major constituents of the essential oils determine their biological properties, although in many cases the biological effect may be different due to synergy among different chemical molecules [37]. To evaluate the effects of *a*-pinene, which is the major component of mastic oil (Table 1), 3 dpf zebrafish larvae were treated for four days with 67.5 and 135 ppm of *a*-pinene, corresponding to five and 10 times higher concentrations than those existing in the natural oil. In contrast to mastic essential oil treatment, larvae exposed to these concentrations of *a*-pinene developed normally and their swimming behavior was physiological. However, regarding the high dose of *a*-pinene, there was a statistically significant reduction in the number of fluorescently labeled neuromasts (37.5 ± 6.4 (S.D) in the treated larvae n = 12 versus 52.5 ± 3.5 (S.D) in the control group n = 12, *p*-value 2.04 × 10^−6^ despite the fact that no morphological defects were observed. These results indicate that mastic essential oil has a unique composition and its properties could be due to a synergy of its constituents, as also suggested by previous studies [16,33].

Essential oils appear as promising candidates of a new generation of products for human and animal nutrition and health, due to their beneficial effects on digestion and gut microbial community [38,39,40]. Concerning mastic essential oil, despite the great number of studies reporting on its different actions [5,6,7,8,16], information about its potential effects at the gene expression level, within the context of dietary supplementation, is lacking. To investigate this, zebrafish were fed with dry food soaked in 2% of mastic essential oil, starting from 5 to 42 dpf. These experimental conditions were based on the digestive system development which starts at 18 h post fertilization (hpf), while food uptake and digestion are functional from 5 dpf onwards. 

We first studied the morphology of the intestine of control and mastic oil fed animals at 42 dpf, using histochemical analysis. Hematoxylin and Eosin (H&E) staining showed normal development of the intestine in both experimental groups (Figure S2a,b). Furthermore, no significant differences were detected in the apoptotic pattern of the intestines of both groups, as assessed by caspase-3 immunostaining (Figure S2c,d). These results indicate that mastic diet did not affect the physiological development of the gastrointestinal system and this is important for the evaluation of gene responses to the mastic oil supplemented diet. 

**Figure 2:**
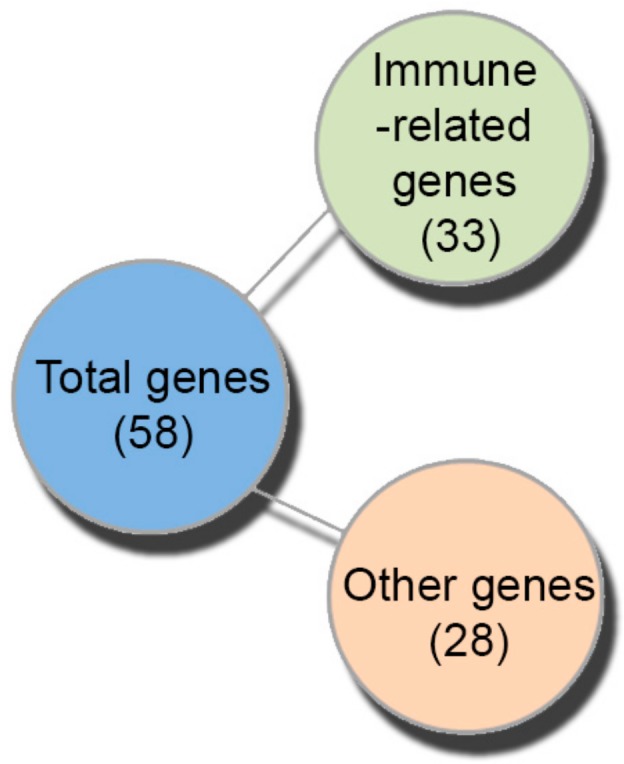
Schematic representation of gene expression data of zebrafish fed with mastic essential oil diet.

Gene expression analysis is a powerful tool to investigate immediate and reversible responses to environmental chemicals and dietary alterations and may provide evidence about the mechanisms and modes of action for several classes of molecules and natural products. To investigate the transcriptional responses to mastic oil diet in zebrafish, mRNA was isolated from 42 dpf treated and control groups and further subjected to cDNA microarray analysis, as described in Materials and Methods. By a selection criteria of fold change ≥ 1.7 and *p*-value < 0.05, 58 genes were identified that displayed differential expression in mastic oil-treated versus the control group (Figure 2). Of these, seven transcripts were up-regulated while 51 were down-regulated (Table 2 and Table S1). Gene ontology characterization [41] showed that 33 differentially expressed genes were related to the immune defense against pathogens (Table 2). Among these genes, a large group was related to the interferons (IFNs), virus-induced cytokines that exhibit the first line of defense against viruses, also exhibiting antiproliferative and immunomodulatory effects [42] (Figure 2 and Table 2). 

**Figure 3:**
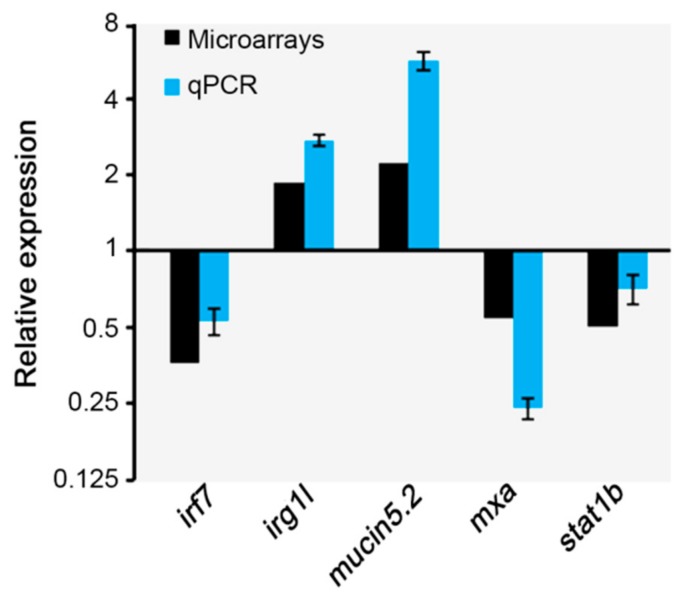
Validation of microarray data by qPCR analysis. Comparison of relative expression differences for *irf7, irg1l*, *mucin5.2*, *mxa*, and *stat1*, determined by microarray and qPCR analysis in zebrafish fed with mastic oil diet. The fold changes in qPCR were normalized to *gapdh* and *rpl13*. Data are expressed as mean ± S.E (*n* = 3).

As shown in Table 2, an immune-related differentially expressed gene was *stat1b*, one of the two *stat1* zebrafish orthologs. STAT proteins induce transcription of IFN-stimulated genes upon binding of type I interferon to its heterodimeric receptor [43]. In addition, *irf7* and *irf1b*, two members of the interferon regulatory factor (IRF) family, have been identified by our microarray screen. These transcription factors are mediators of early inflammatory genes in infected cells [44,45]. Among the differentially expressed genes were also *interferon-induced protein 44* and *interferon stimulated gene 12* that encode interferon-induced proteins which are involved in the protective response to viral infection [46]. Other genes affected by the mastic oil diet belong to the family of the very large interferon inducible GTPases (VLIG) that also contribute to the host cellular response to IFNs [47]. Along this line, *mx* gene that encodes Mx protein, a member of the large GTPases family, known to be exclusively induced by IFNs [48], was also down-regulated (Table 2). Changes in the expression levels of the genes described above were validated by qPCR analysis (Figure 3).

It is well established that IFNs functions are complicated and in many cases the production of IFNs interferes with antibacterial mechanisms [49]. The expression pattern of IFN-related transcripts in the mastic oil group might be indicative of a protective mechanism against bacteria. In support to this hypothesis, the microarray analysis revealed two up-regulated genes, *irg1l* and *muc 5.2* (Table 2, Figure 3) that play important roles in the host defense against bacterial pathogens. 

*Irg1* gene encodes for the *cis*-aconitate decarboxylase that catalyzes the decarboxylation of *cis*-aconitate, an intermediate of the tricarboxylic acid cycle, to produce itaconic acid. This metabolic derivative exhibits important function towards bacteria, such as *Salmonella enterica* and *Mycobacterium tuberculosis,* by enhancing the production of reactive oxygen species (ROS) within macrophage-lineage cells [50]. Notably, Irg1 protein is localized in mitochondria, a cellular compartment recently recognized as a platform orchestrating anti-inflammatory and immune cell defense mechanisms [51]. In light of the above findings, it is tempting to speculate that *irg1* may be a mediator of the antibacterial function of mastic oil, which is known since ancient times and documented by several previous studies [5,6,7,8,16]. 

*Muc 5.2* encodes a protein that belongs to the mucin family of o-glycosylated glycoproteins which form long and fibrilar polymers secreted by specialized goblet cells. Mucins regulate the production of mucus, which is composed by a complex mixture of glycoproteins, water, macromolecules, electrolytes, and cells that covers the gastrointestinal epithelium [52]. Mucus is important for several functions, including protection of the epithelium from bacteria and pathogens [52,53]. Low amounts of mucins lead to increased susceptibility to infections of the oral cavity and the gut epithelium. Interestingly, recent studies have shown that the maturation and function of the mucus layer are strongly influenced by the gut microbiota, and in return, mucins can regulate the production of mucosa-associated bacteria [54,55]. Like humans, the gut microbiota of zebrafish is influenced by nutritional, microbiological and environmental factors and plays important roles in host metabolism and immune function [56]. 

Our data provide new perspectives for understanding the effects of mastic essential oil diet depicting (i) down-regulation of genes of the IFN system and (ii) immunometabolic actions through induction of *irg1l* and increased expression of *muc5.2.* These results, combined with existing knowledge on the protective effects of mastic on the gastrointestinal tract and oral cavity health [5,6,7,8,16], open up the possibility that mastic essential oil in animal and human diet, may exert a prebiotic-like effect on the intestinal environment by altering the amount and composition of secretions produced by the intestinal mucosa. These changes could modulate both the physical and chemical properties of the intestinal environment and facilitate the digestion and absorption of nutrients, making it challenging to dissect these mechanisms, especially in humans. Subsequent biochemical and genomic experiments based on our observations are required to enable elucidation of the underlying mechanisms by which mastic essential oil influences metabolism and immune system function. Our work encourages future in vivo research focusing on the actions of mastic essential oil on host microbial dynamics and immunity.

## 3. Materials and Methods

### 3.1. Zebrafish Maintenance

Zebrafish were maintained in an automated circulating system, equipped with appropriate filters to ensure good water quality and a healthy aquatic environment. Room and tank water temperature were set at 28 ± 0.5 °C and the photoperiod was 14 h light: 10 h dark. Fish were fed with commercially dry food twice a day. Breeding and embryo collection were performed under standard conditions and before sacrifice, animals were anaesthetized in 0.016% Tricaine MS-222. Zebrafish were staged according to Kimmel, et al. [57] and reared in compliance with the internationally accepted principles for laboratory animal use and care as found in the European Community guidelines (EEC Directive of 1986; 86/609/EEC) and the Recommended Guidelines for Zebrafish Husbandry conditions (https://www.eufishbiomed.kit.edu/59.php). The present study was performed according to international, national, and institutional rules considering animal experiments, clinical studies and biodiversity rights. All experimental protocols described in this study were approved by the FORTH Ethics Committee. 

### 3.2. Extraction and Analysis of Mastic Essential Oil

Commercial mastic gum was supplied by the Chios Mastic Gum Growers Association. In Chios island the resinous gum is collected by hand directly from the plant or from the ground underneath. The air dried parts were milled by hand in VIORYL laboratories and then the use of an inhouse experimental distillation equipment was applied. The mastic oil was produced by a direct dry distillation of the mastic gum under vacuum. The temperature ranged from 50 to 110 °C. The sample was centrifuged and the upper layer was separated. No further fractionation took place. GC/MS analysis was carried out in a GC-MS (GC: 6890A, Agilent Technologies, USA, MSD: 5973, Agilent Technologies) using a Factor Four VF 1 ms column (25 m, 0.2 mm i.d., 0.33 μm film thickness, Agilent Technologies). 0.1 μL of essential oil was directly injected and a 1:100 split ratio was applied. The oven temperature was set at 50 °C for 1 min, followed by a temperature gradient of 2.5 °C/min. When temperature reached 160 °C it was kept steady for 20 min. Then, a step of 50 °C/min was applied until oven temperature was 250 °C, where it was kept for 15 min. Helium was used as carrier gas with a flow rate of 1 mL/min. Injector and transfer line temperatures were set to 200 and 250 °C, respectively. The mass spectrometer operated in the electron impact mode with the electron energy set to 70 eV. Volatiles identification was completed according to mass spectra comparison to Willey/NIST 0.5 and in-house created libraries (VIORYL S.A.).

### 3.3. Exposure of Zebrafish Larvae to Mastic Oil and In Vivo Staining of Lateral Line Neuromasts

Mastic essential oil (density 0.86 g/mL) was diluted in absolute ethanol at a concentration of 5% *v/v*. At 3 dpf, larvae were placed in 6-well plates (10 fish in 5 mL of system water per well) and exposed to mastic essential oil for four days at different concentrations (10–200 parts per million, ppm, corresponding to 10–200 mg/L). The solutions of mastic essential oil were renewed at 24-h intervals. The ethanol concentrations corresponding to 10, 20, 100, and 200 ppm were 0.0235%, 0.047%, 0.235%, and 0.47%, respectively and did not cause any toxic effects. The control group was treated with system water containing equivalent concentrations of ethanol (vehicle control). The mortality of the larvae was recorded during the assay.

Lateral line neuromasts were assessed for mechanosensitivity by in vivo staining with the vital fluorescent dye Synaptogreen C4 (Biotium), also known as FM1-43. Following a four-day exposure to mastic oil or *a*-pinene, 7 dpf larvae were rinsed twice in E3 medium (5 mM NaCl, 0.17 mM KCl, 0.33 mM CaCl_2_, and 0.33 mM MgSO_4_) and incubated with 3 μM Synaptogreen C4 for 30 s at room temperature, under dark conditions. Larvae were then rinsed three times in E3 medium, anaesthetized in 0.016% Tricaine MS-222 and fluorescently labeled neuromasts were counted bilaterally, under a fluorescent microscope. Three independent experiments were performed for each concentration of mastic essential oil.

### 3.4. Preparation of Mastic Oil Supplemented Fish Food

Dietary supplementation of zebrafish food with mastic essential oil (2% *v/v*), was prepared by dissolving 0.2 mL of essential oil in 10 mL of 100% ethanol and subsequent addition of the mastic oil/ethanol solution to 10 g of commercial dry food. Removal of ethanol was effected by evaporation until the food was completely dry. Zebrafish were divided into separate groups based on their diet. For a period of 38 days, starting at 5 dpf, zebrafish larvae were fed with 2% *v/v* mastic oil supplemented diet or control diet consisting of commercial dry food. Over the course of the experiments, zebrafish individuals subjected to the mastic oil diet were observed for any morphological or behavioral changes. At 42 dpf, zebrafish were sacrificed for further analyses.

### 3.5. Whole Mount Immunofluorescence

Seven dpf larvae were fixed in 4% PFA for 2 ⅟_2_ h at room temperature. After fixation, larvae were washed twice with PBS, transferred to 100% methanol, and stored at −20 °C. Acetylated tubulin staining was performed as previously described [58]. 

### 3.6. H&E Staining 

Zebrafish were fixed for 1 day in 10% formalin and then dehydrated in graded concentrations of ethanol, xylol, and finally embedded in paraffin. Serial 3 μm whole body sagittal sections were prepared, mounted on slides, and stained with hematoxylin and eosin.

### 3.7. Immunohistochemistry

Immunostaining was performed on formalin-fixed, paraffin-embedded tissue sections by the streptavidin-biotin peroxidase labeled (LSAB) method. Pretreatment of the sections in microwave oven was performed. Sections were pretreated with 10 mM sodium citrate buffer pH 7.0 and then incubated with anti-cleaved caspase-3 antibody (Cell Signaling, 1:1000) for 12 h. Negative controls were incubated and processed identically with omission of the primary antibody.

### 3.8. RNA Preparation and cDNA Synthesis

Total RNA was extracted from control and mastic oil-treated zebrafish at the stage of 42 dpf (three biological replicates per sample). RNA isolation and purification were performed using the RNeasy Mini Kit (Qiagen, Hilden, Germany) with DNase to eliminate genomic DNA contamination. The concentration and quality of the purified RNA samples were determined by Nanodrop spectrophotometer (Thermo Scientific, Waltham, MA, USA) and verified by use of the Agilent 2100 Bioanalyzer (Agilent Technologies, Santa Clara, CA, USA). One microgram of purified total RNA was used as a template for reverse transcription and cDNA synthesis with the PrimeScript RT Reagent Kit with gDNA Eraser (Takara, Saint-Germain-en-Laye, France).

### 3.9. Microarray Analysis

The transcriptomic profile of control and mastic oil-treated zebrafish was analyzed by microarrays, using the Affymetrix Platform of the GeneCore Facility in EMBL (Heidelberg, Germany). RNA samples were processed, labeled, and hybridized to the Zebrafish Gene 1.0 ST Array (Affymetrix, Santa Clara, CA, USA), according to standard Affymetrix protocols, as described [59]. Raw data were normalized, processed, and visualized with the GeneSpring software (Agilent Technologies) [59]. The list of genes was generated from the final data by *p*-value sorting ≤ 0.05 and absolute differential expression ≥ 1.7 fold change. Microarray data have been deposited to the Gene Expression Omnibus (GEO) database under the accession number GSE74098.

### 3.10. Quantitative Real-Time PCR (qPCR)

Quantitative real-time PCR reactions were performed on a CFX96 Touch Real-Time PCR Detection System (Bio-Rad Laboratories, Inc., CA, USA) using the KAPA SYBR^®^ FAST qPCR Master Mix Universal Kit (KAPA Biosystems) reagent. Three biological replicates were run in technical triplicates. The reaction mixture (10 μL) was composed of 5 μL of qPCR Master Mix (2x), 0.25 μL of each primer (10 μΜ), 2 μL of diluted cDNA template (12-fold), and 2.5 μL of Nuclease-free water. The qPCR cycling conditions were initial denaturation at 95 °C for 3 min followed by 40 cycles of 95 °C for 3 s, 60 °C for 30 s, and 72 °C for 15 s. *Gapdh* and *rpl13* (geNorm kit, PrimerDesign) were used as internal controls. Normalization and relative quantification of gene expression were performed with the qBase Plus software (Biogazelle). Primers for the genes of interest were designed based on *Danio rerio* nucleotide sequences found in NCBI, their Tm was 60 °C and their sequences are listed in Table 3.

### 3.11. Statistical Analysis

Statistical analysis was performed using the 2-tailed Student’s *t* test considering *p* ≤ 0.05 as statistical significance.

## Figures and Tables

**Table 1:** Composition of mastic essential oil. The semi-transparent mastic resin was obtained as a trunk exudate from the mastic tree. Mastic essential oil was extracted from the mastic resin by direct gum distillation and analyzed by GC/MS. RT: Retention time; MW: Molecular weight.

Compounds	RT	Relative (%) Area	Formula	Structure	MW
*α*-pinene	9.24	67.71	C_10_H_16_	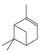	136.24
camphene	9.73	0.70	C_10_H_16_	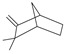	136.24
verbenene	9.87	0.07	C_10_H_14_	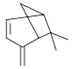	134.22
*β*-pinene	10.93	3.05	C_10_H_16_	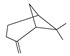	136.24
myrcene	11.79	18.81	C_10_H_16_	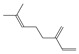	136.24
limonene	13.44	0.89	C_10_H_16_	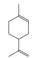	136.24
linalol	16.67	0.73	C_10_H_18_O	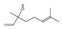	154.25
*α*-campholenic ald	17.60	0.26	C_10_H_16_O	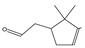	152.23
pinocarveol	18.61	0.32	C_10_H_16_O	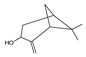	152.23
*trans*-verbenol	18.78	0.07	C_10_H_16_O	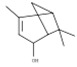	152.23
*cis*-verbenol	18.97	0.69	C_10_H_16_O	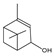	152.23
verbenone	21.59	0.32	C_10_H_14_O	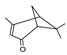	150.22
caryophyllene	33.61	0.50	C_15_H_24_	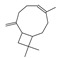	204.36

**Table 2:** Differentially expressed immune-related genes in zebrafish upon dietary supplementation with mastic essential oil. Genes were categorized by a selection criteria of fold change ≥ 1.7 and *p*-value < 0.05 in treated *versus* control zebrafish. FC (abs): Fold Change (absolute).

Gene Symbol	Gene Description	NCBI Gene ID	*p-*Value	FC (abs)
*muc5.2*	mucin 5.2	572172	9.37 × 10^−3^	2.24
*irg1l*	immunoresponsive gene 1, like	562007	1.56 × 10^−2^	1.87
*LOC100001880*	interferon-induced very large GTPase 1-like, si:dkey-202l22.6	100001880	1.50 × 10^−3^	1.92
*LOC556554*	skin mucus antibacterial l-amino acid oxidase-like//orthologous to human interleukin 4 induced 1	556554//337166	1.03 × 10^−4^	−1.96
*irf7*	interferon regulatory factor 7	393650	2.14 × 10^−4^	−2.80
*irf1b*	interferon regulatory factor 1b	792160	4.02 × 10^−4^	−1.77
*si:dkey-79f11.7*	si:dkey-79f11.7 (interferon-induced protein 44)	100007552	1.70 × 10^−2^	−1.88
*LOC795887*	interferon-induced protein 44	795887	6.45 × 10^−4^	−2.10
*zgc:152791*	zgc:152791 (interferon stimulated gene 12)	641326	3.38 × 10^−5^	−2.13
*LOC558511*	orthologous to human interferon alpha inducible protein 27 like 2	558511	2.16 × 10^−3^	−1.80
*LOC556241*	interferon-induced very large GTPase 1-like, si:dkey-85k7.12	556241	2.62 × 10^−4^	−2.03
*il21r.1*	interleukin 21 receptor, tandem duplicate 1	100134976	1.17 × 10^−2^	−1.76
*b2m*	beta-2-microglobulin	30400	6.20 × 10^−4^	−2.21
*Fas*	Fas cell surface death receptor (TNF receptor superfamily, member 6)	768248	8.53 × 10^−4^	−1.70
*hla-dpa1*	HLA-DPA1 protein, zgc:123107	553497//641415	1.41 × 10^−2^	−1.83
*Mxa*	myxovirus (influenza) resistance A	360142	2.20 × 10^−3^	−1.84
*stat1b*	signal transducer and activator of transcription 1b	368481	3.73 × 10^−5^	−1.99
*urgcp, LOC100005729*	upregulator of cell proliferation	100005729	7.42 × 10^−3^	−1.82
*LOC798269*	tumor necrosis factor receptor superfamily member 5	798269	1.22 × 10^−2^	−1.85
*lgals3bpa*	lectin, galactoside-binding, soluble, 3 binding protein a	677742	1.44 × 10^−3^	−2.48
*lgals3bpb*	lectin, galactoside-binding, soluble, 3 binding protein b	405809	1.15 × 10^−4^	−1.91
*lgals9l1*	lectin, galactoside-binding, soluble, 9 (galectin 9)-like 1	337597	4.33 × 10^−2^	−1.98
*ccl38a.5| ccl38a.1*	chemokine (C-C motif) ligand 38, duplicate 5| chemokine (C-C motif) ligand 38, duplicate 1	563208| 794050	3.81 × 10^−3^	−2.59
*ccl34b.4*	chemokine (C-C motif) ligand 34b, duplicate 4	556621	2.59 × 10^−2^	−2.09
*LOC100334176*	GTPase IMAP family member 8	100334176	2.33 × 10^−2^	−3.02
*LOC100150889, tapbpl*	TAP binding protein- like	100150889	3.27 × 10^−3^	−1.74
*LOC100007087*	basic leucine zipper ATF transcriptional factor -like, si:dkey-23i12.7	100007087	8.18 × 10^−3^	−2.19
*ftr19*	finTRIM family, member 19 (tripartite motif-containing protein 47)	100301514	3.72 × 10^−4^	−1.74
*si:ch211-284p22.1, trim35-19*	tripartite motif containing 35-19	562814	2.74 × 10^−5^	−1.81
*crp3*	C-reactive protein 3	100141350	3.68 × 10^−3^	−2.96
*LOC100332428| LOC562648*	poly [ADP-ribose] polymerase 14	100332428| 562648	1.42 × 10^−3^	−1.72
*zgc:153893, xaf1*	zgc:153893, (XIAP-associated factor 1)	767709	2.52 × 10^−4^	−1.75
*gig2d* *, LOC100005232*	grass carp reovirus (GCRV)-induced gene 2d	100005232	1.20 × 10^−4^	−1.88

**Table 3:** List of primers used for qPCR analysis.

Gene Symbol	Gene ID	Forward Primer	Reverse Primer
*irf7*	393650	GTGGAAAGTGGGCAGTACGA	GCCGCTGACTATAGCCCATT
*irg1l*	562007	TGCACTAGATGTGGCAGAGC	AGCATACATGTGCTGGCAGT
*mucin 5b*	572172	GGTGTCTGTTCCGATCAATC	TCATCCTTGTCGCCATTGTA
*mxa*	360142	TCGAGTTTCGACCTTGGCAC	GACGCTTGCTTGCAATTGTGTA
*stat1b*	368481	GCTTATCCCGAGATACACTCCT	GCTGCTTTACGTGGCATTTC

## Supplementary Materials

The following are available online at https://www.mdpi.com/1420-3049/24/21/3919/s1. Figure S1: GC/MS analysis of mastic essential oil. Figure S2: Histochemical staining and apoptotic pattern of zebrafish intestine upon mastic oil diet. Table S1: Other differentially expressed genes in zebrafish upon dietary supplementation with mastic essential oil.
