# Supplementary material for: Effects of the Essential Oil from Pistacia lentiscus Var. chia on the Lateral Line System and the Gene Expression Profile of Zebrafish (Danio rerio)
Source: Molecules. 2019 Oct 30;24(21):3919. doi: 10.3390/molecules24213919 (PMC6864543; doi:10.3390/molecules24213919)
Supplement: Supplementary file 1 [file molecules-24-03919-s001.zip › Supplementary files/Figure S1.docx]

**Figure S1**


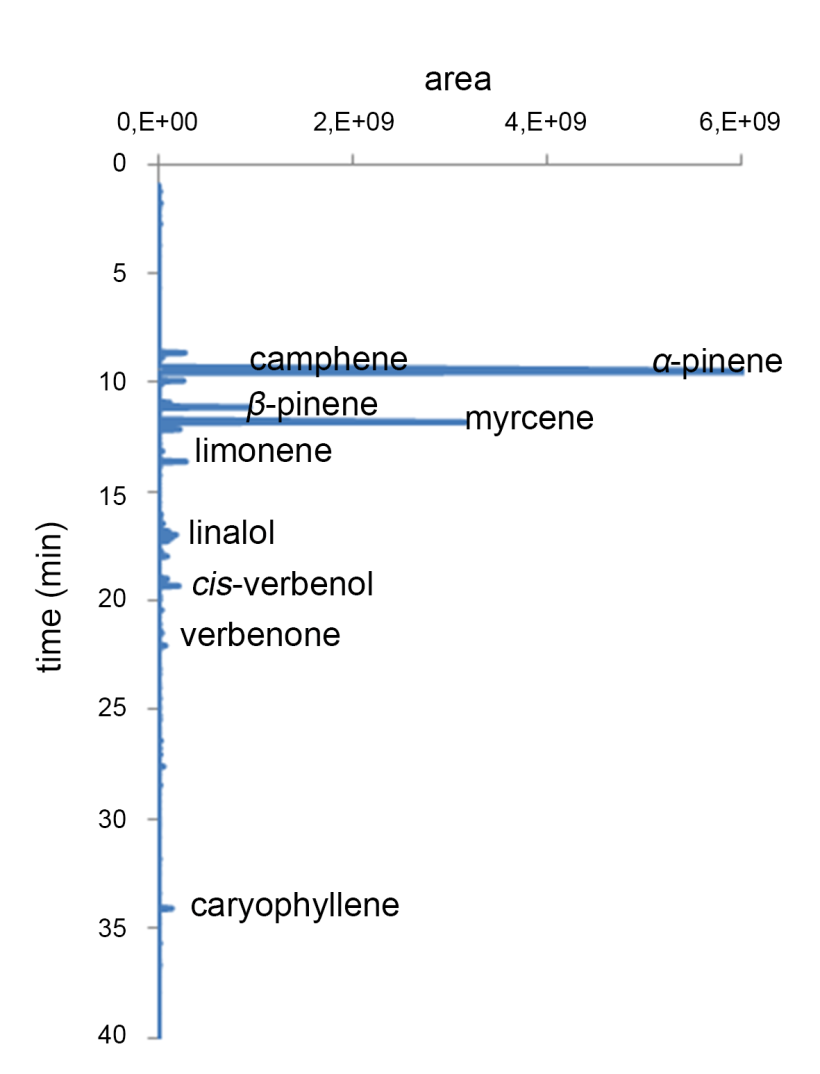


**Figure S1.** GC/MS analysis of mastic essential oil. Volatiles identification was performed with the use of known retention times (where analytical standards were available) and the simultaneous use of mass spectra from in house (VIORYL SA) and commercial libraries (Willey/NIST 0.5).
