# Supplementary material for: Effects of the Essential Oil from Pistacia lentiscus Var. chia on the Lateral Line System and the Gene Expression Profile of Zebrafish (Danio rerio)
Source: Molecules. 2019 Oct 30;24(21):3919. doi: 10.3390/molecules24213919 (PMC6864543; doi:10.3390/molecules24213919)
Supplement: Supplementary file 1 [file molecules-24-03919-s001.zip › Supplementary files/Figure S2.docx]

**Figure S2**


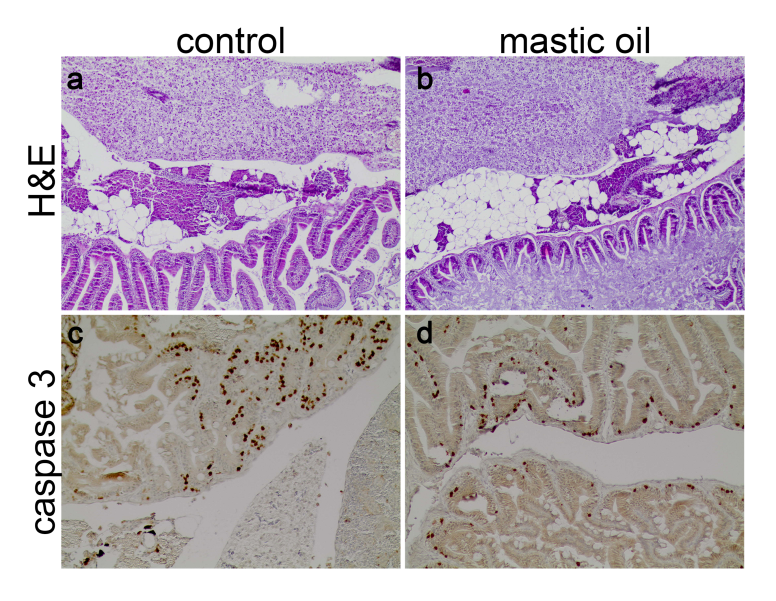


**Figure S2.** Histochemical staining and apoptotic pattern of zebrafish intestine upon mastic oil diet. Zebrafish were fed with dry food soaked in 2% of mastic oil from the stage of 5 to 42 dpf. (a, b) Serial 3 μm whole body sagittal sections mounted on slides were stained with Hematoxylin and Eosin. (c, d) Immunostaining for cleaved caspase-3 was performed on formalin-fixed, paraffin-embedded tissue sections by the streptavidin-biotin peroxidase labeled (LSAB) method (×200).
