# Supplementary material for: Effects of the Essential Oil from Pistacia lentiscus Var. chia on the Lateral Line System and the Gene Expression Profile of Zebrafish (Danio rerio)
Source: Molecules. 2019 Oct 30;24(21):3919. doi: 10.3390/molecules24213919 (PMC6864543; doi:10.3390/molecules24213919)
Supplement: Supplementary file 1 [file molecules-24-03919-s001.zip › Supplementary files/Table S1.docx]

**Table S1**

**Table S1.** Other differentially expressed genes in zebrafish upon dietary supplementation with mastic essential oil. Genes were categorized by a selection criteria of fold change ≥ 1.7 and *p*-value < 0.05 in treated *versus* control zebrafish. FC (abs): Fold Change (absolute).

| **Gene Symbol** | **Gene Description** | **NCBI Gene ID** | ***p-*value** | **FC (abs)** |
| --- | --- | --- | --- | --- |
| *stxbp4, LOC567916* | syntaxin binding protein 4-like | 567916 | 1.47 x 10^-2^ | 1.85 |
| *prss60.1, zgc:162180* | serine protease 60.1 | 799770 | 2.74 x 10^-2^ | 1.74 |
| *krt1-19d* | keratin, type 1, gene 19d | 664718 | 1.15 x 10^-2^ | 1.70 |
| *LOC567193* | similar to human galectin-4, uncharacterized protein si:dkey-151j17.4 | 567193 | 2.50 x 10^-2^ | 2.38 |
| *ddx43\|LOC557043* | DEAD (Asp-Glu-Ala-Asp) box polypeptide 43 \| hypothetical LOC557043 | 100136871// 557043 | 6.08 x 10^-4^ | −4.60 |
| *LOC100001181* | tripartite motif protein TRIM29-like | 100001181 | 4.87 x 10^-3^ | −1.81 |
| *LOC100006656* | si:ch1073-188e1.1 | 100006656 | 1.15 x 10^-4^ | −2.40 |
| *LOC100007087* | si:dkey-23i12.7, basic leucine zipper transcriptional factor ATF-like | 100007087 | 8.18 x 10^-3^ | −2.19 |
| *LOC100330813* | tripartite motif-containing protein 47-like | 100330813 | 1.23 x 10^-3^ | −1.98 |
| *LOC564808* | uncharacterized LOC564808 | 564808 | 3.12 x 10^-3^ | −3.24 |
| *LOC795959* | gamma-glutamyltranspeptidase 1, si:dkey-222h21.12 | 795959 | 1.12 x 10^-3^ | −1.98 |
| *LOC798013* | carbohydrate (chondroitin 4) sulfotransferase 12-like, si:dkey-26i13.7 | 798013 | 1.56 x 10^-2^ | −2.53 |
| *LOC798358* | membrane-spanning 4-domains, subfamily A, member 4-like | 798358 | 4.59 x 10^-3^ | −1.97 |
| *ogfrl2, si:busm1-6a2.1* | si:busm1-6a2.1, opioid growth factor receptor-like 2 | 368512 | 1.81 x 10^-3^ | −2.32 |
| *si:ch211-114l13.9* | si:ch211-114l13.9 | 796649 | 1.45 x 10^-2^ | −1.85 |
| *si:ch211-217k17.10* | si:ch211-217k17.10 | 798704 | 2.90 x 10^-2^ | −2.16 |
| *si:ch211-244b2.3* | si:ch211-244b2.3 uncharacterized protein LOC557230 | 557230 | 8.34 x 10^-5^ | −2.04 |
| *si:ch211-245h14.1* | si:ch211-245h14.1 uncharacterized protein LOC563420 | 563420 | 5.01 x 10^-4^ | −1.71 |
| *si:dkey-58f10.13* | uncharacterized protein si:dkey-58f10.13 | 100149772 | 5.22 x 10^-3^ | −1.81 |
| *zgc:152658* | zgc:152659 [Orthologous to human genes including GIMAP4 (GTPase, IMAP family member 4)] | 767632 | 8.51 x 10^-5^ | −1.89 |
| *zgc:152670* | zgc:152670, prolyl 4-hydroxylase alpha II-like | 557059 | 2.88 x 10^-3^ | −3.91 |
| *zgc:162182* | uncharacterized protein LOC100038769; zgc:162182 [orthologous to human OGFR] | 100038769 | 3.09 x 10^-4^ | −1.84 |
| *zgc:171506* | zgc:171507, uncharacterized protein LOC792896 | 792896 | 1.87 x 10^-4^ | −1.82 |
| *casp3, zgc:171731* | caspase 23, apoptosis-related cysteine peptidase | 563034 | 3.09 x 10^-4^ | −1.83 |
| *cx30.9* | connexin 30.9 | 402821 | 3.08 x 10^-4^ | −2.26 |
