# Supplementary figures and images for: Effects of the Essential Oil from Pistacia lentiscus Var. chia on the Lateral Line System and the Gene Expression Profile of Zebrafish (Danio rerio)
Source: Molecules. 2019 Oct 30;24(21):3919. doi: 10.3390/molecules24213919 (PMC6864543; doi:10.3390/molecules24213919)

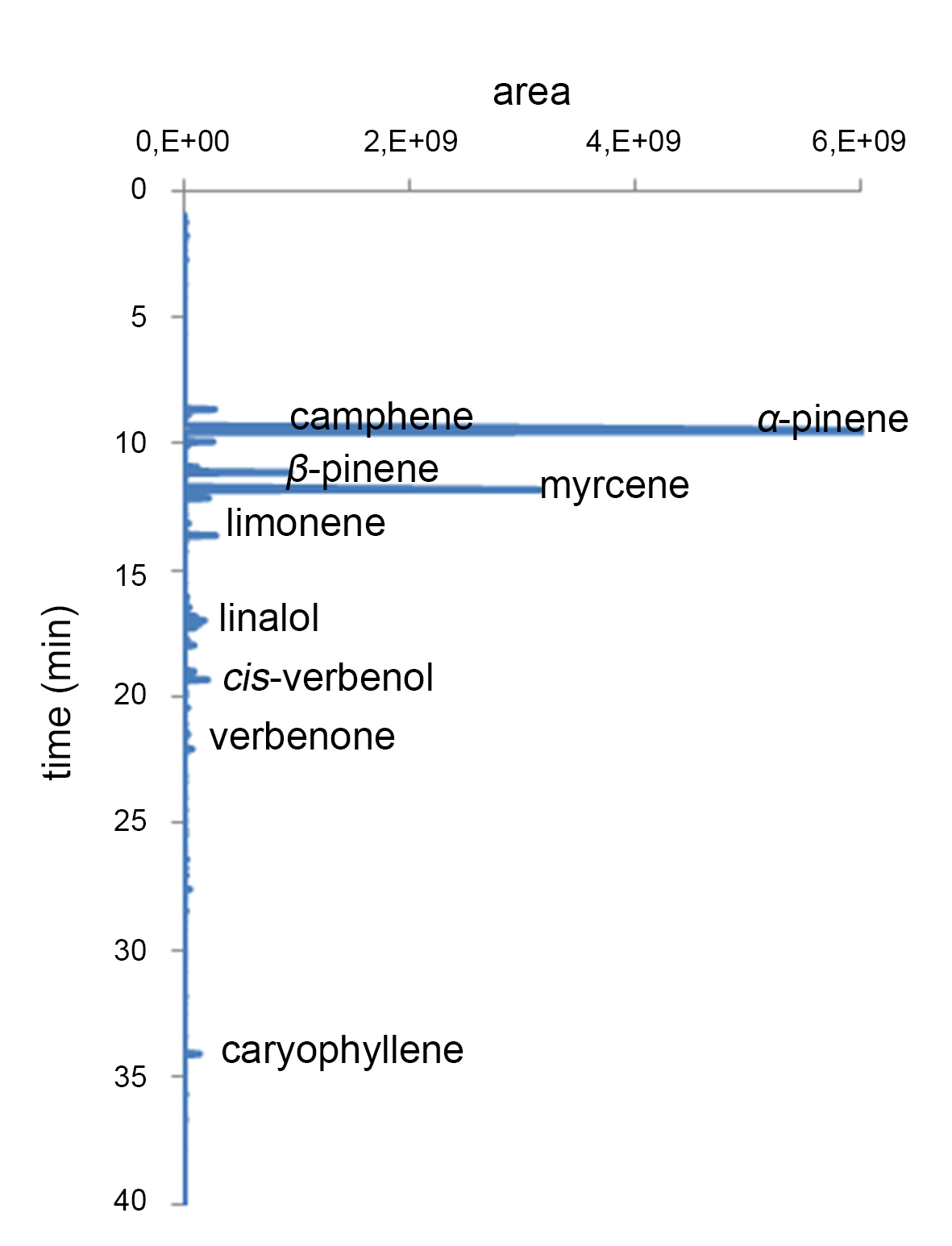

Supplement: Supplementary file 1 [file molecules-24-03919-s001.zip › Supplementary files/FIGURE S1.tif]

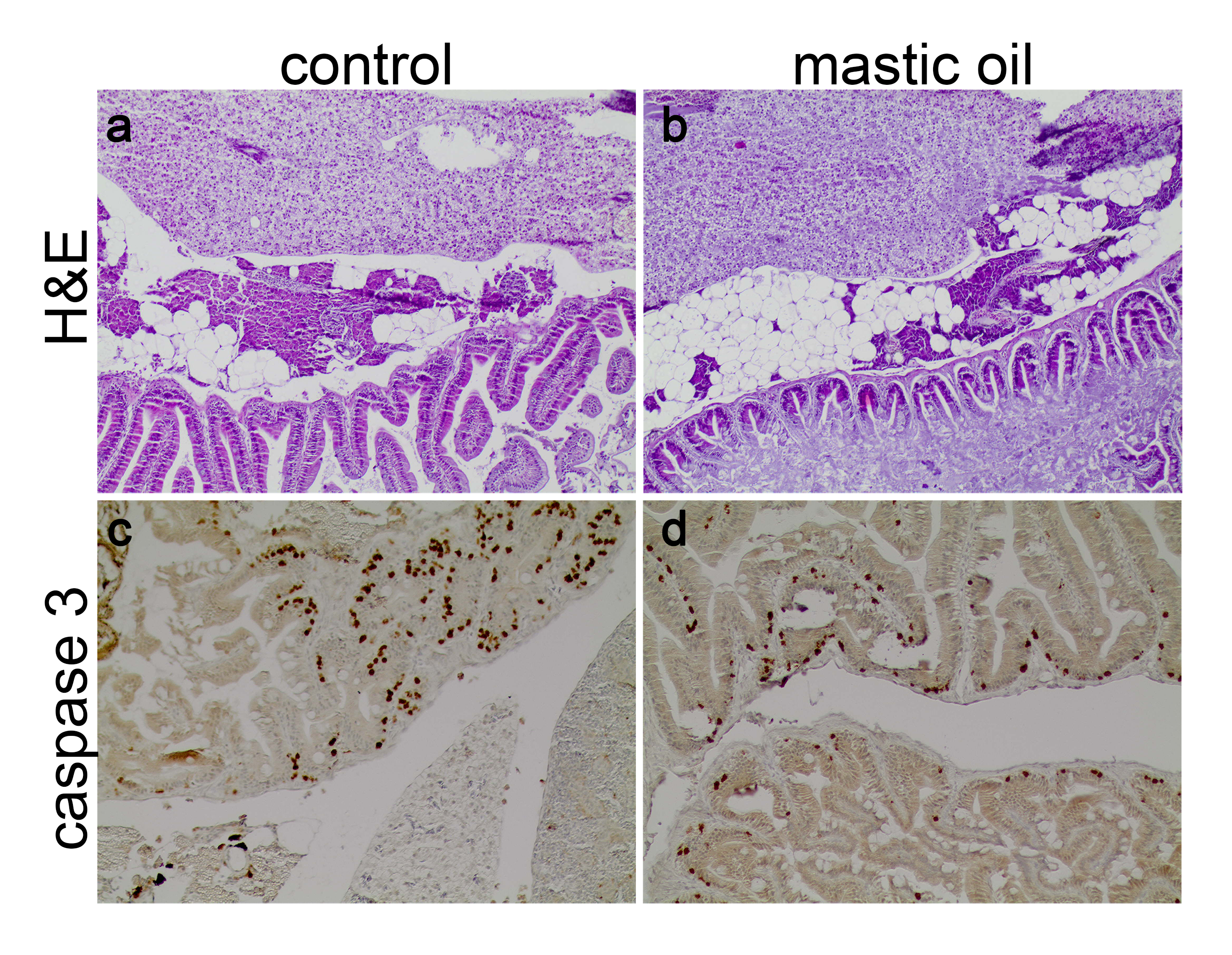

Supplement: Supplementary file 1 [file molecules-24-03919-s001.zip › Supplementary files/FIGURE S2.tif]
